# Supplementary material for: Downregulation of mitochondrial metabolism is a driver for fast skeletal muscle loss during mouse aging
Source: Commun Biol. 2023 Dec 8;6:1240. doi: 10.1038/s42003-023-05595-3 (PMC10709625; doi:10.1038/s42003-023-05595-3)
Supplement: Supplementary file 2 — Supplementary Information [file 42003_2023_5595_MOESM2_ESM.pdf]

Supplementary figures and Supplementary Tables S6 and S6

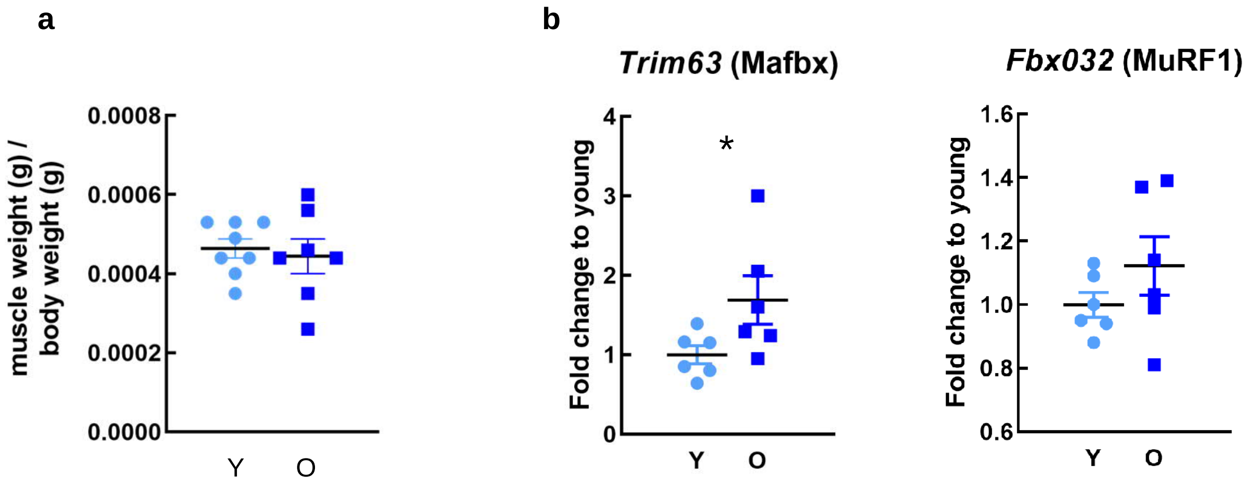

Fig. S1 Aged EDL shows molecular damage but no loss of muscle mass. (a) The graphic represents the sarcopenic index (muscle weight/body weight) of both young and old EDL muscle. (b) mRNA expression levels of two markers of muscle damage, Trim63 and Fbx032 in young and old EDL (Y, young; O, old). The samples are relative to young controls (n=6 per group). Statistical significance is given as follows *p<0.05 (Mann-Whitney U test).

Supplemental figure 1. Raquel Fernando, Anastasia V. Shindyapina, Mario Ost, Didac Santesmasses, Yan Hu, Alexander Tyshkovskiy, Sun Hee Yim, Jürgen Weiss, Vadim N. Gladyshev, Tilman Grune, José Pedro Castro

scRNA-seq analyses reveal downregulation of mitochondrial-related processes in aged fast skeletal muscle cells

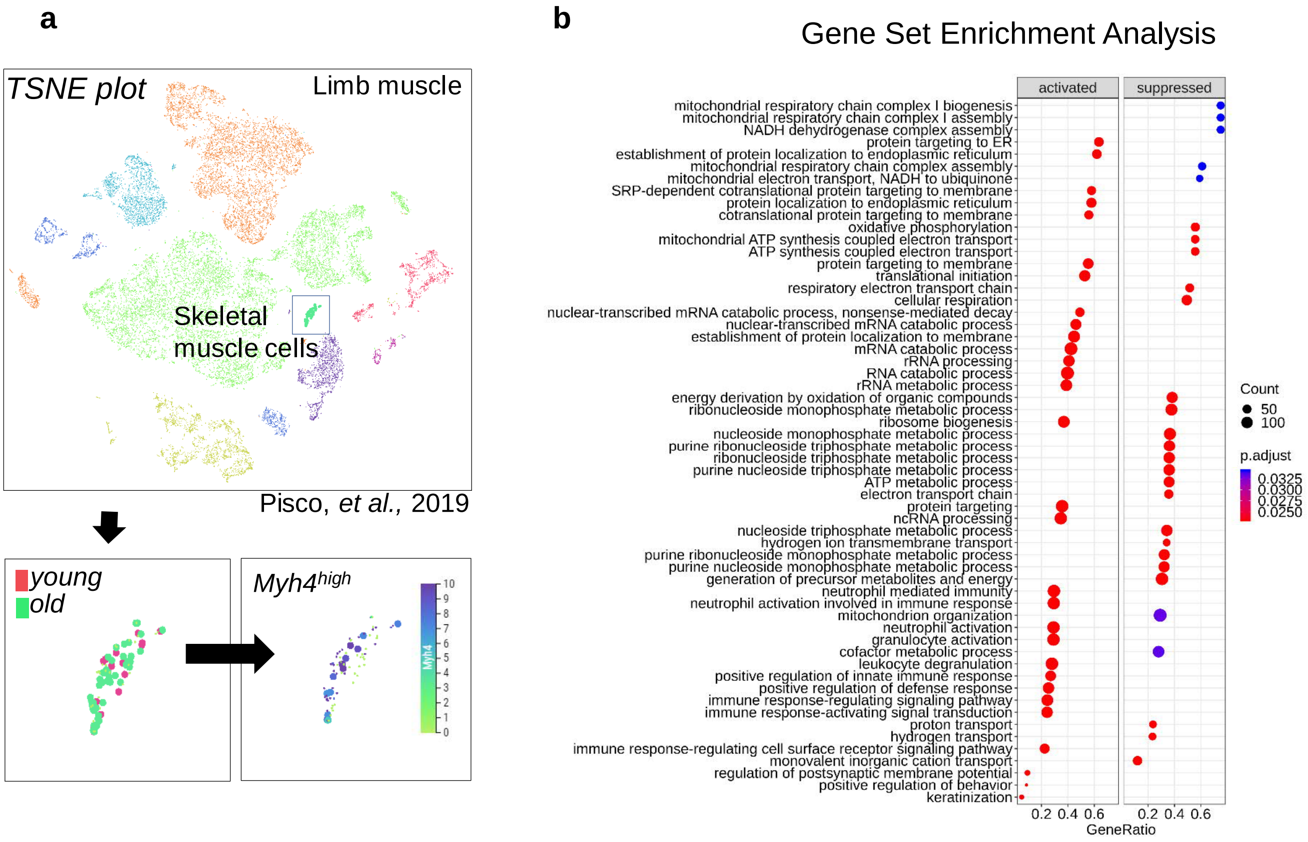

Fig. S2. scRNA-seq analyses reveal downregulation of mitochondrial-related processes in aged fast skeletal muscle cells. (a) Workflow diagram: TSNE plot of mice limb muscle cells from publicly available data. Skeletal muscle cells were selected and sorted by age (young or old). Only Myh4 positive cells (Myh4^{high}) were sorted for further analysis. (b) GSEA of genes present in Myh4^{+}. The left side shows the biological processes that are activated and on the right the ones which are suppressed.

Supplemental figure 2. Raquel Fernando, Anastasia V. Shindyapina, Mario Ost, Didac Santesmasses, Yan Hu, Alexander Tyshkovskiy, Sun Hee Yim, Jürgen Weiss, Vadim N. Gladyshev, Tilman Grune, José Pedro Castro

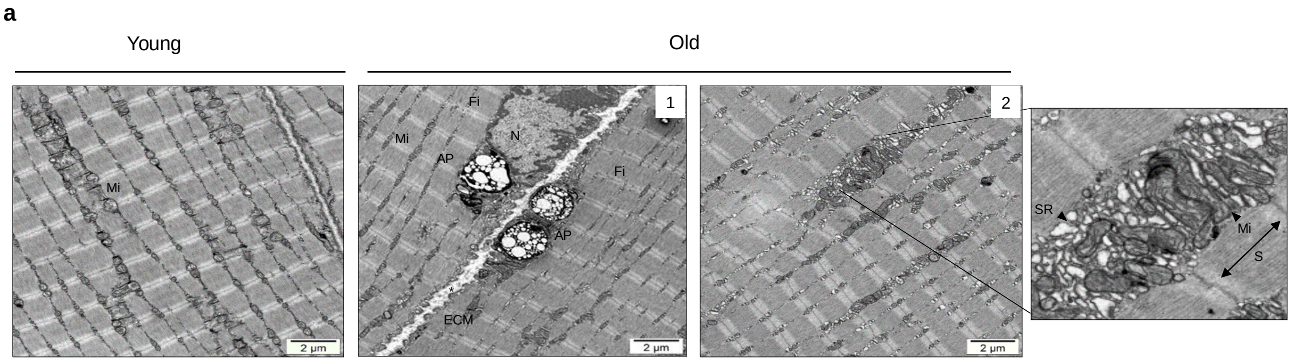

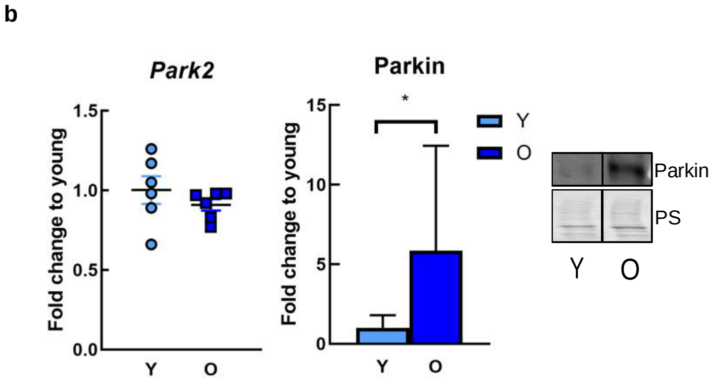

Fig. S3. Aged EDL fibers show damaged mitochondria possibly due to impaired mitophagy. (a) Representative electron microscopy images of young and old EDL muscle. Few characteristics are depicted, numbered and zoomed in (n=3 per group). (b) The left graphic shows the mRNA levels of Park2 and on the right the protein levels of Parkin (normalized to ponceau S (PS)) (n=6). Both are relative to young. Statistical significance is given as follows *p<0.05 (Mann-Whitney U test).

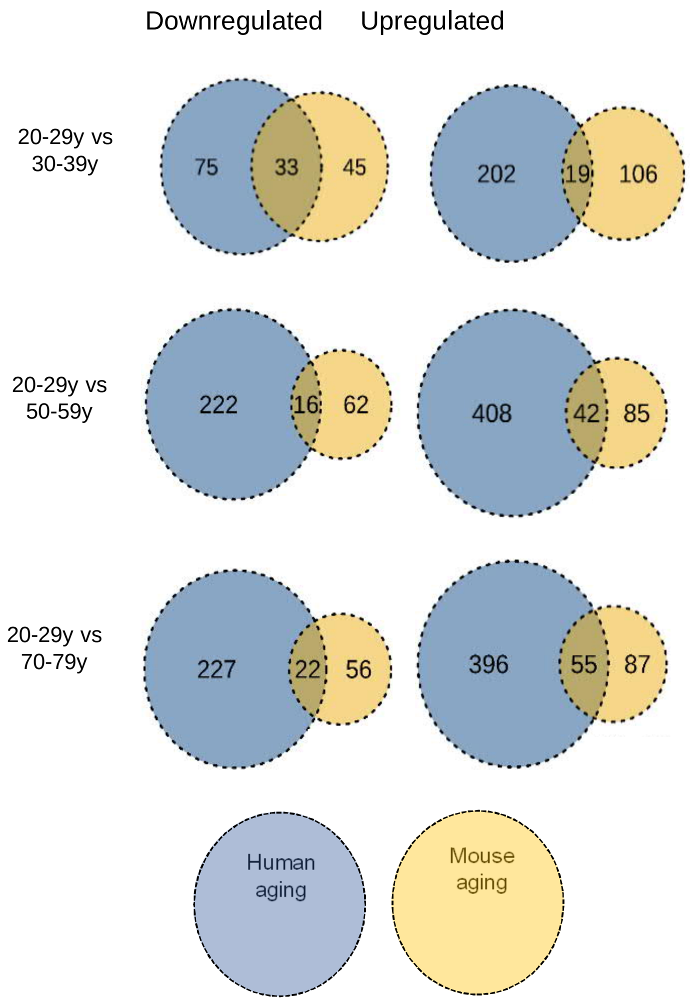

Fig. S4. Overlap of genes significantly up- and downregulated from mouse and human in response to aging. On the left, the Venn diagrams represent the downregulated genes in mouse (compared to young) and human aging (across different human ages, compared to a younger group, 20-29y), as well as the ones which overlap between both species. On the right, the Venn diagrams represent the upregulated genes from mouse and human aging.

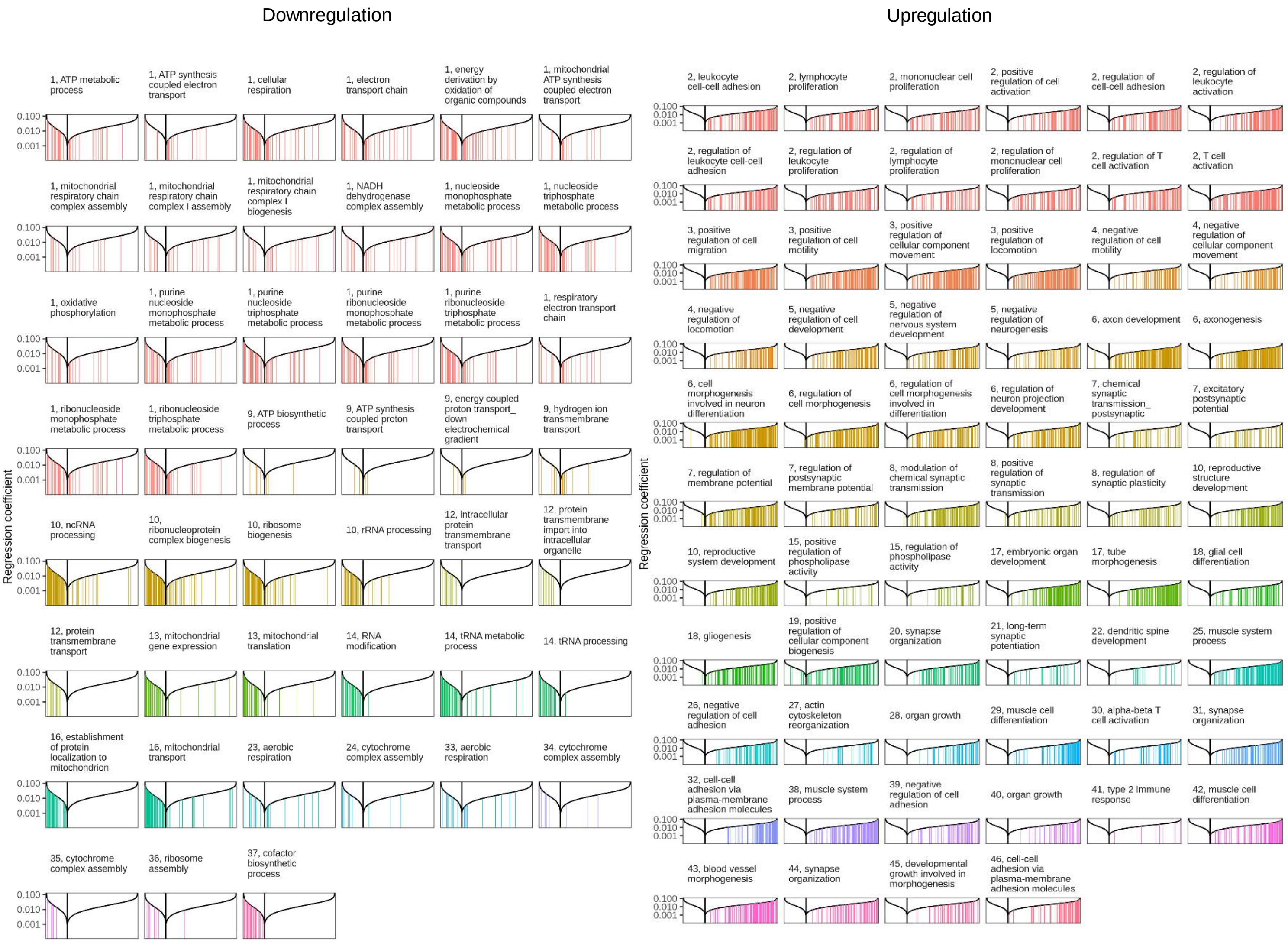

Fig. S5. Up and downregulated biological processes associated with aging in EDL muscle. On the left panel, the graphics represent several different downregulated biological processes associated with aging. On the right, the graphics represent biological processes associated with aging.

Supplementary Tables S5 and S6

Supplemental Table 5 - List of primers

Primer	Forward	Reverse
Myh7	GCCTGGGCTTACCTCTCTATCAC	CTTCTCAGACTTCCGCAGGAA
Myh2	CAGCTGCACCTTCTCGTTTG	CCCGAAAACGGCCATCT
Myh1	GGACCCACGGTCGAAGTTG	CCCGAAAACGGCCATCT
Myh4	CAATCAGGAACCTTCGGAACAC	GTCCTGGCCTCTGAGAGCAT
Trim63	ATGCACACTGGTGCAGAGAG	TGTAAGCACACAGGCAGGTC
Fbx032	GCTGGTGGAAAACATCATTGACAT	CATCGGGTGGCTGCCTTT
Park2	AAGAAGACCACCAAGCCTTGTC	CAAACCAGTGATCTCCCATGC
Tbp	CTGGAATTGTACCGCAGCTT	ATGATGACTGCAGCAAATCG
Actin	CACTGCCGCATCCTCTTCCT	GATTCCATACCCAAGAAGGAAGGC
Rpl13a	GTTCGGCTGAAGCCTACCAG	TTCCGTAACCTCAAGATCTGCT

Supplemental Table 6 - List of primary and secondary antibodies

Primary antibody	Dilution	Secondary antibody	Dilution
MAFbx (F-9) (Santa Cruz)	1:1000	IRDye® 800CW goat anti-mouse IgG	1:20000
Parkin (Prk8) (Cell Signaling)	1:1000	IRDye® 800CW goat anti-mouse IgG	1:20000
