# Supplementary material for: Downregulation of mitochondrial metabolism is a driver for fast skeletal muscle loss during mouse aging
Source: Commun Biol. 2023 Dec 8;6:1240. doi: 10.1038/s42003-023-05595-3 (PMC10709625; doi:10.1038/s42003-023-05595-3)
Supplement: Supplementary file 3 — Description of Supplementary Materials [file 42003_2023_5595_MOESM3_ESM.docx]

**Description of Additional Supplementary Files**

**File name:** Supplementary Data

**Description:** Numerical source data for all graphs in the manuscript

**File name:** Table S1

**Description:** EDL genes ranked list.

**File name:** Table S2

**Description:** List of the metabolites significantly altered in aged EDL muscle.

**File name:** Table S3

**Description:** List of the total metabolites and their ratio (old/young)

**File name:** Table S4

**Description:** List of functions enriched by at least one signature

**File name:** Supplementary Material

**Description:** Uncropped blots related to supplementary figures.
