# Supplementary material for: Downregulation of mitochondrial metabolism is a driver for fast skeletal muscle loss during mouse aging
Source: Commun Biol. 2023 Dec 8;6:1240. doi: 10.1038/s42003-023-05595-3 (PMC10709625; doi:10.1038/s42003-023-05595-3)
Supplement: Supplementary file 9 — Supplementary Material [file 42003_2023_5595_MOESM9_ESM.pdf]

Supplementary figures

Data Availability

Relative to Fig1D – data availability

Membrane 1

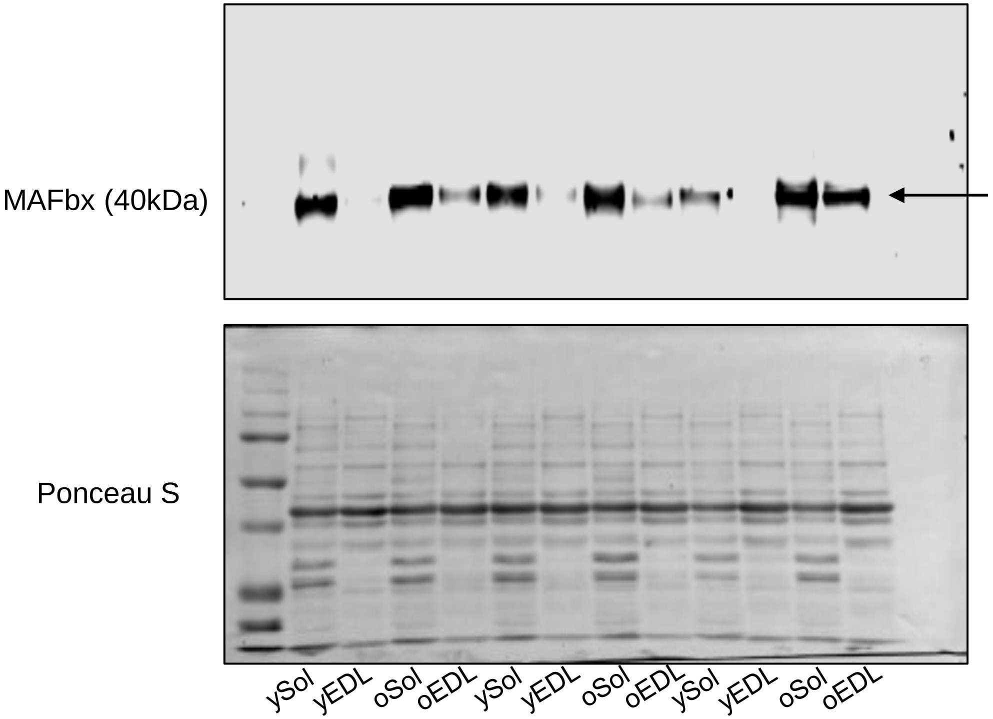

Membrane 2

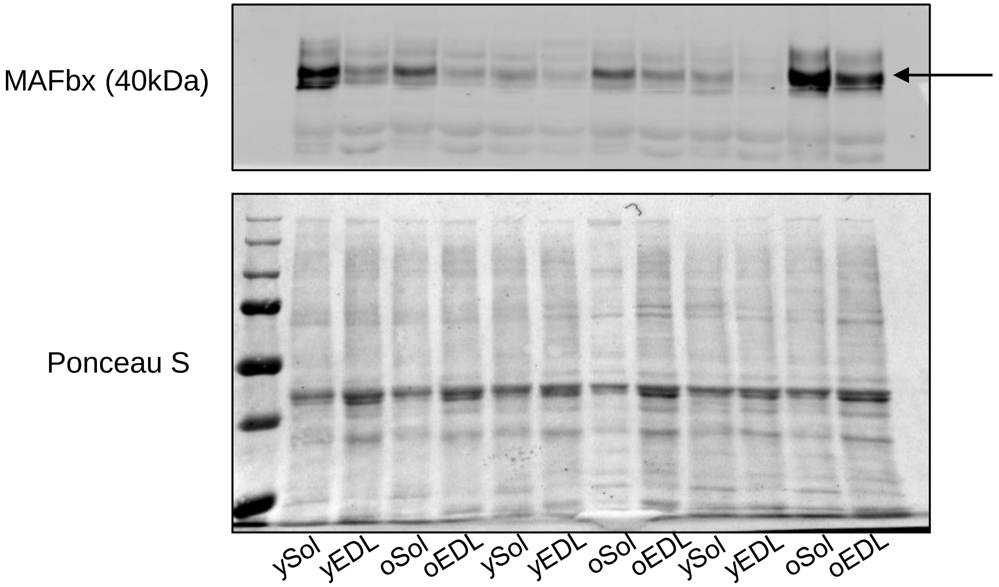

y/oSol – young/old *Soleus* muscle
y/oEDL – young/old *Extensor digitorum longus*

Supplementary figures

Data Availability

Relative to FigS3 B – data availability

Membrane 1

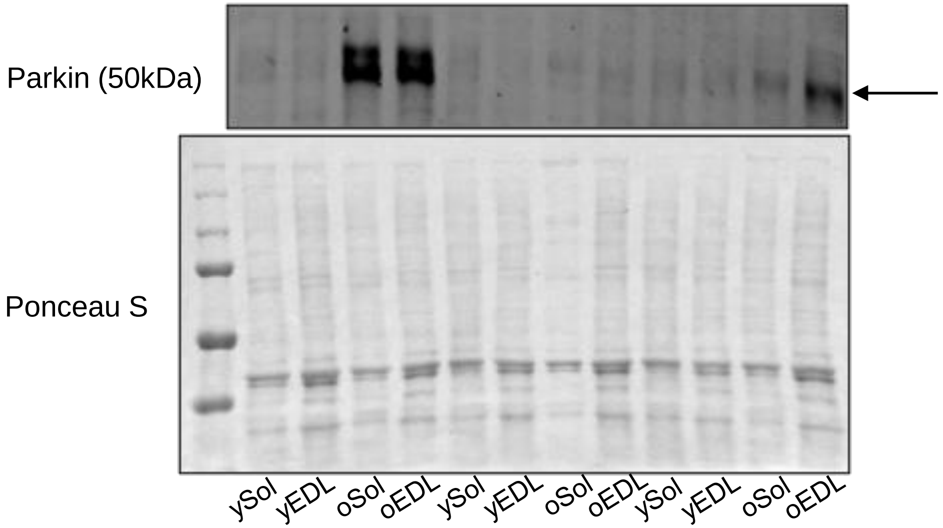

Membrane 2

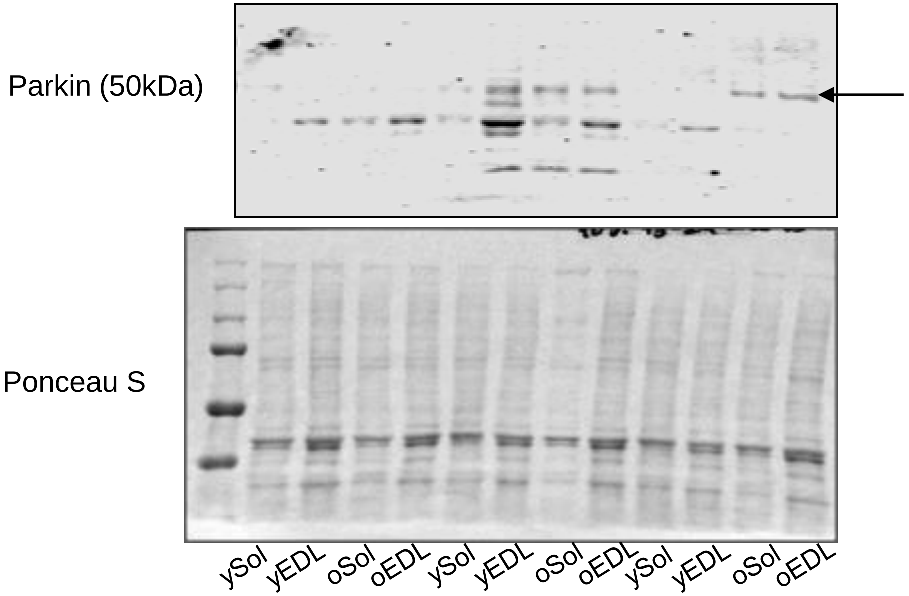

y/oSol – young/old *Soleus* muscle
y/oEDL – young/old *Extensor digitorum longus*
